# Evaluation of saliva as a complementary technique to the diagnosis of COVID-19: a systematic review

**DOI:** 10.4317/medoral.24424

**Published:** 2021-02-20

**Authors:** Katherine Sagredo-Olivares, Constanza Morales-Gómez, Juan Aitken-Saavedra

**Affiliations:** 1 Undergraduate, Faculty of Dentistry, University of Chile, Santiago, Chile; 2 Department of Oral Pathology and Medicine, Faculty of Dentistry, University of Chile, Santiago, Chile; 3 Graduate Program in Dentistry, School of Dentistry, Federal University of Pelotas, Pelotas, Brazil; 4 Dental Service, San Camilo Hospital, San Felipe, Chile

## Abstract

**Background:**

Infectious disease coronavirus 2019 (COVID-19) is caused by the SARS-CoV-2 virus, and it mainly affects the upper respiratory tract. The gold standard for its diagnosis is real-time reverse transcription polymerase chain reaction (RT-qPCR) performed on a nasopharyngeal swab. In contrast, testing saliva has significant advantages as a diagnostic method.

**Material and Methods:**

We searched for articles evaluating saliva as a diagnostic method for COVID-19 on the PUBMED/MEDLINE, WEB OF SCIENCE, COCHRANE, and SCIELO platforms. We initially found 233 articles and 20 were selected for inclusion following the Preferred Reporting Items for Systematic Reviews and Meta-Analyses protocol: 18 cross-sectional studies and 2 case reports, including 8 from America, 8 from Asia, and 4 from Europe. The studies evaluated the presence of viral RNA, IgG, IgM, and IgA in samples of unstimulated saliva from adults with confirmed or suspected COVID-19. The vast majority of the studies performed RT-qPCR on the saliva samples and compared the results with the gold standard (a nasopharyngeal swab of the same patient).

**Results:**

Saliva samples analyzed by RT-qPCR, reverse transcription isothermal amplification (RT-LAMP), spectroscopy, and enzyme-linked immunosorbent assay (ELISA) offer high sensitivity to detect SARS-CoV-2 in the early stages of the disease and among asymptomatic patients as compared to nasopharyngeal swab RT-qPCR. In addition, the self-collection of saliva offers the possibility of receiving telemedicine instructions to carry out the test, reducing the risk of contagion.

**Conclusions:**

The diagnosis of COVID-19 through saliva is sensitive, non-invasive, and is of low risk for the healthcare professionals. However, further studies are recommended to validate its clinical use.

** Key words:**Saliva, SARS-CoV, COVID19, Salivas.

## Introduction

Coronaviruses are a type of RNA virus that belong to the Coronoviridae family and that have been detected in humans and other animals. Infectious coronavirus diseases can cause severe acute respiratory syndrome coronavirus (SARS-CoV). In late 2019, in Wuhan, China, cases of pneumonia were identified that were caused by a virus initially called the 2019 novel coronavirus (2019-nCoV), but it was then renamed “severe acute respiratory syndrome coronavirus 2” (SARS-CoV-2). This virus rapidly spread worldwide (1). As of July 2020, it is estimated that there had been a total of 17.6 million confirmed cases and 680 thousand deaths from COVID-19 worldwide (2). SARS-CoV-2 mainly affects the upper respiratory tract, and its most common symptoms include fever, cough, dyspnea, and myalgia. However, sputum production, headache, hemoptysis, and diarrhea have also been reported (1). As a result, patients diagnosed with coronavirus-19 (COVID-19) suffer from physical and psychological deterioration, affecting their health-related quality of life (3).

For the diagnosis of this disease, the gold standard applies reverse transcriptase polymerase chain reaction (RT-qPCR) to samples obtained using nasopharyngeal and oropharyngeal swabs (4). The specificity of this testing method appears to be high, but false-positive results have been detected due to swab contamination, especially in asymptomatic patients. The sensitivity of the technique is estimated to be around 66%–80% (4).

Due to the progressive increase in positive cases, there is great interest in improving the SARS-CoV-2 diagnostic method, to achieve a greater specificity and sensitivity, and allowing for a larger number of infected people to be diagnosed more rapidly, including asymptomatic patients, since it has been reported that they also spread the virus (5). In addition, taking a nasopharyngeal swab causes discomfort and the possibility of bleeding for the patient, and a risk of contagion for health professionals collecting the sample. In this context, saliva could offer an alternative, since it has been shown to be useful for diagnosing different respiratory diseases, is easy to collect and store, and its collection is non-invasive (6). Because the SARS-CoV-2 infection mechanism begins with binding to the receptor for angiotensin-converting enzyme 2 (ACE2), which is widely expressed in the salivary glands, the virus is likely to be detecTable in saliva (7,8). The global SARS-CoV-2 pandemic has unleashed a global public health problem. The massive level of contagion and the large percentage of deaths that this virus has produced has prompted the research and development of new detection techniques. Therefore, the objective of this systematic review was to evaluate saliva as a diagnostic technique for COVID-19.

## Material and Methods

- Protocols and sources of information

This systematic review was conducted according to the guidelines of the Cochrane Handbook for Systematic Reviews of Interventions, following the four-phase flow diagram of the Preferred Reporting Items for Systematic Reviews and Meta-Analyses Statement. This review is registered in the International Prospective Register of Systematic Reviews PROSPERO with the code: CRD42020209856. The literature search was carried out by two independent reviewers in July 2020. The following databases were screened: PubMed (National Library of Medicine), Cochrane (Elsevier), and Web of Science (Thomson Reuters). In addition, the reference lists of the selected articles were searched manually for additional articles. Articles published in English, Spanish, and Portuguese over the last 2 years were considered. The search strategy is described in Supplement1 (http://www.medicinaoral.com/medoralfree01/aop/24424_supplement1.pdf). Two researchers used a predefined data collection form to independently extract and summarize the data from the included studies (K.A.S.O and C.B.M.G). The adjudicating senior author resolved any disagreements (J.P.A.S). The main outcome was an evaluation of saliva as a diagnostic fluid among people infected by COVID-19.

- Data extraction

We analyzed all studies reported in English, Portuguese or Spanish that met the inclusion criteria/PICO. We extracted information such as the authors’ names, the country where the study was undertaken, the publication year, study type, factors reported, sample size, the sensitivity, and specificity of the salivary diagnostic methods (RT-qPCR, RT-LAMP, spectroscopy and enzyme-linked immunosorbent assay (ELISA) as compared to the gold standard method (RT-qPCR of a nasopharyngeal swab) and the percentage of positivity in the diagnosis of COVID-19 through salivary diagnosis. The authors used Review Manager software to synthesize the results according to the Cochrane Collaboration statistical guidelines. A random-effects model was used. Data were gathered in spreadsheets and divided into qualitative data and quantitative data. Qualitative data were synthesized through a narrative review. Due to the high degree of heterogeneity in terms of the different studies and methodologies, conducting a quantitative meta-analysis was considered inappropriate.

## Results

**Figure 1:**
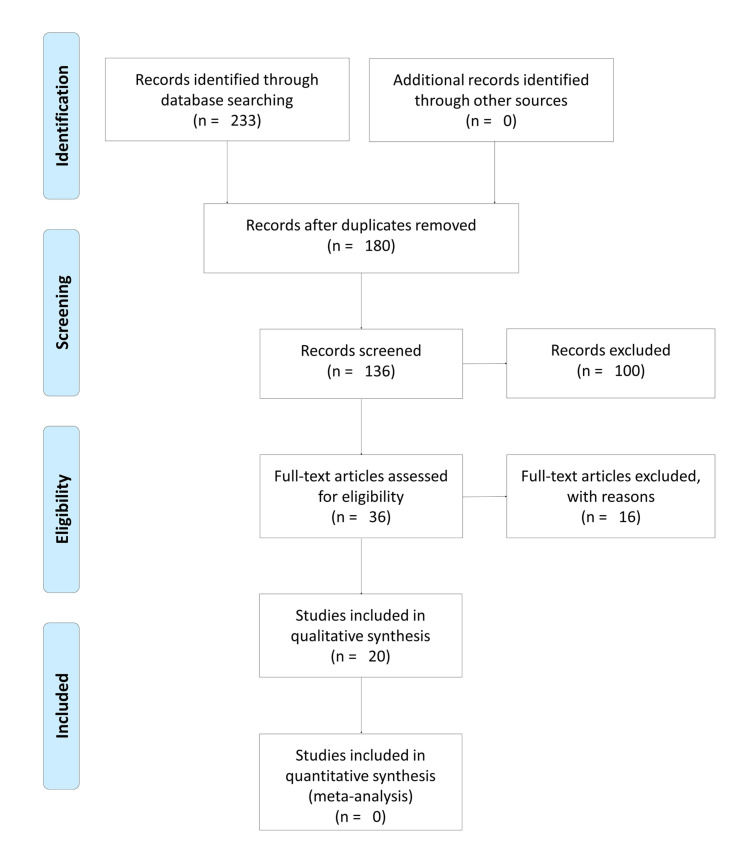
Search flow according to the Prisma statement.

A total of 233 references were identified in the 4 electronic databases. None were identified through other sources. After the removal of 53 duplicates and the use of a 5-year filter, 136 titles/abstracts were examined and 36 articles met the eligibility criteria and were included for full-text review. After a thorough reading of these articles and the exclusion of those that contained insufficient information, 20 articles were finally included for analysis (9-28). The flow chart of the study is presented in Fig. 1.

All articles were published in English in 2020. There were 17 cross-sectional studies, 2 case reports and 1 prospective study, including 8 studies from America, 8 from Asia, and 4 from Europe. Considering all 20 selected articles, a total of 2,355 patients were examined after obtaining diagnostic samples but only 1,557 patients from these 20 articles were characterized into subgroups before the examination. Within these non-additive subgroups, a total of 335 were confirmed (21.5%) and 1177 were suspected (75.6%) of having COVID-19, and within these 2 groups, 179 were categorized as symptomatic (11.5%) and 112 as asymptomatic (7.2%).

Regarding the salivary sample collection method, 13 articles (65%) used patient self-collection samples, 5 articles (25%) had the sample collected by a professional, and in 2 articles (10%) who collected the sample was not specified. In addition, 3 articles used professional instruction through telemedicine to guide the self-collection of samples. Among the 20 select articles, 15 articles collected, in addition to saliva, a nasopharyngeal swab for a direct comparison, while the other 5 studies used the patient’s initial diagnostic data as the diagnostic reference standard. Regarding the molecular biology diagnostic technique, one or more techniques were evaluated. Among the 20 selected articles, 18 articles processed the salivary sample by using RT-qPCR and 2 articles only used an immunoassay (ELISA, for example). Among the studies that performed RT-qPCR, four articles included in the methodology an extraction of viral RNA using the QIAamp Viral RNA Mini Kit (Qiagen). In addition, 9 articles compared RT-qPCR with other techniques such as RT-LAMP, ST, LFA-based RST, nCoV-DK, SMGNP, or HT-LAMP. These data are summarized in Table 1.

**Table 1:**
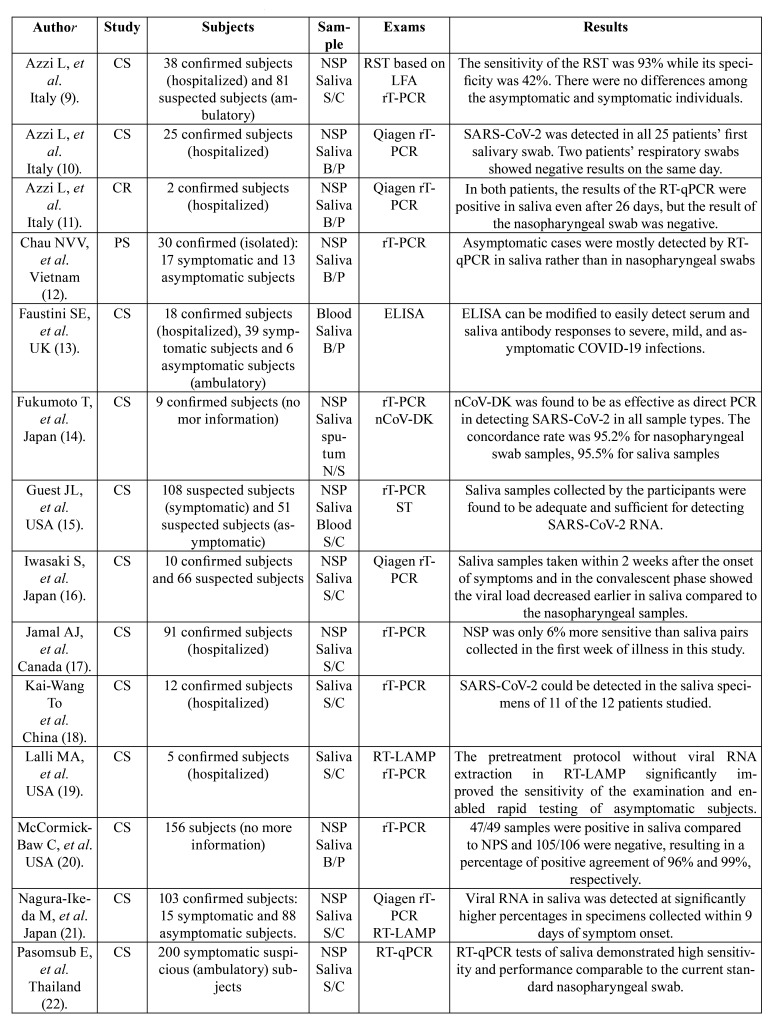
Characterization of the articles of the systematic review.

**Table 1 cont.**
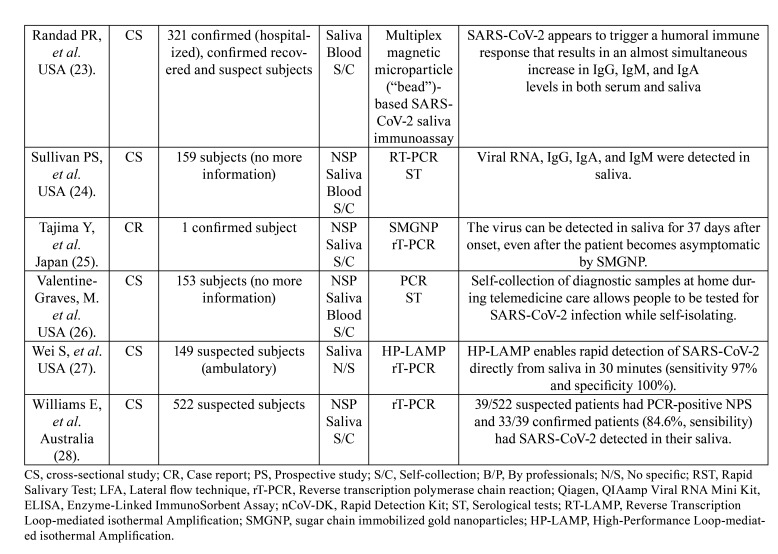
Characterization of the articles of the systematic review.

## Discussion

Due to the need to optimize the diagnostic method to achieve a high specificity and sensitivity and to allow a greater number of infected people to be diagnosed more rapidly, this review compiled information that evaluated saliva as an alternative testing sample for the diagnosis of COVID-19. Reviewing the geographical distribution of the selected studies, we can observe that there was an equality between the number of cases globally and the number of publications per continent, being America (17.3 million), Asia (7.6 million), and Europe (6.4 million) among the locations with the largest number of confirmed cases around the world (2). It is interesting to note that the selected articles came from countries with a high level of scientific production, such as the United States, Japan, and China.

Regarding the method of collecting the samples, the articles all pointed out the self-collection of saliva as an efficient technique, being easy to collect, non-invasive, and with very little risk of contagion among healthcare professionals (19). In contrast to saliva, the nasopharyngeal swab technique can be uncomforTable, in addition to presenting a risk of bleeding in patients with thrombocytopenia (25). In some studies, they also collected blood and saliva samples, where equivalent immunoglobulin levels could be observed, and they concluded that saliva could be a tool to evaluate short and long-term humoral immunity in COVID-19 infections, and for understanding the nature of the natural and vaccine-induced responses to this disease (13,22). It is interesting to mention that within the selected studies, three articles instructed their patients through telemedicine to perform a self-collection of samples from their homes, a methodology that, considering the current context and the importance of social distancing, reinforces the relevance of its use and implementation (15,23,26).

Among the selected studies, RT-qPCR was the most widely used test to diagnose COVID-19 in saliva. This test demonstrated a sensitivity of 84.2% and a specificity of 98.9% compared to the nasopharyngeal swab RT-qPCR results. In addition, a 97.5% concordance rate was recorded between both samples (22). Furthermore, some articles used Qiagen to extract the viral RNA from saliva samples (10,11,16,21), and they found the RT-qPCR was positive for up to 26 days after the onset of symptoms (11). Other articles evaluated the saliva by means of LAMP, demonstrating that it is a technique that allows for the rapid and sensitive detection of SARS-CoV-2, offering the possibility of easily evaluating the results by colorimetry (19,21). Furthermore, Wei *et al*. observed that with HP-LAMP, positive results can be obtained from saliva in just 30 minutes, observing a sensitivity and specificity of 97% and 100%, respectively (27). One study showed that nCoV-DK is able to effectively detect SARS-CoV-2 in saliva, comparing it to PCR of a nasopharyngeal swab. In addition, it was observed that the detection time could be reduced by half, since the purification and extraction steps of viral RNA are not necessary (14). Also, IgG, IgM, and IgA antibodies were detected in saliva samples by immunoassays in people with severe, mild, and asymptomatic COVID-19 infections, demonstrating that these tests could serve to evaluate short-term and long-term humoral immunity (13,23,24). It is of great importance to highlight that most of the articles mentioned the need to detect asymptomatic people with contagion capacity to control the spread of the pandemic. For this reason, it is urgent to find a diagnostic method that is faster, but just as sensitive and specific, as the gold standard (4). This is why some studies mention that saliva plays an important role in detecting the virus and, through efficient diagnostic tests, the spread of SARS-CoV-2 by asymptomatic patients could be prevented (10-13,19,25). However, other studies affirm that in the samples of asymptomatic patients or convalescent patients, the sensitivity of the tests decreases (21) or is still unknown (22).

Science has focused efforts in recent years to search for and evaluate the effectiveness of salivary biomarkers that can be used to assess the presence, developmental risk and responses to therapies of some diseases (29). In this sense, saliva can play an important role in the transmission of COVID-19 infection, and it also allows for a convenient and non-invasive method of diagnosis (30). The fact that saliva can be used as an alternative to serum for the detection of organic biomarkers is due to the fact that the vast majority of proteins present in the serum are also expressed in saliva (31). It has been indicated that IgG in saliva, for example, may be a useful surrogate marker of this antibody status in serum (32). The COVID-19 virus in the saliva could come from the salivary glands through the ducts or gingival fluid or simply through secretions from the lower and upper respiratory tracts that combine with the saliva (30). Because the SARS-Cov2 infection mechanism begins with binding to the angiotensin-converting enzyme receptor 2 (ACE2), which is widely expressed in the salivary glands, in addition to allowing the detection of the virus in saliva (7,8), stresses the diagnostic role of this fluid in the early stages (33) and it would therefore be of great use in detecting asymptomatic infected patients. Also, it was demonstrated the presence of spike viral protein in salivary glands, reinforcing the theory of saliva as tool to diagnose COVID-19 (34).

Within the limitations of this systematic revision, we can mention that not all of the articles described their patients epidemiologically, which, although it may not be decisive for the purposes of our study, because the virus has been distributed homogeneously in the worldwide population, this could introduce a bias due to differences of viral behavior in saliva due to the age or sex of those infected. What, according to our criteria, corresponds to a true limitation, is that some articles did not report whether the evaluated patients were confirmed or suspected cases, if they only suffered from symptoms, or if they were hospitalized. We believe that characterizing the patients participating in these studies is of vital importance in order to have prior knowledge of the percentage of confirmed patients and to establish the sensitivity and specificity of the diagnostic technique that is being evaluated. Furthermore, not all studies compared their salivary virus detection techniques with the gold standard. This point is essential to evaluate their efficiency as a diagnostic method and for potential follow-up of infected patients. Another limitation is that some studies used saliva samples self-collected by the patients, not specifying whether they were instructed in detail as to how to perform the procedure, so it is not really known if these samples were taken correctly or if the amount collected was sufficient to perform the analysis. Finally, the selected studies used different diagnostic salivary testing methodologies, so they cannot be compared with each other, making it difficult to fully evaluate the literature.

Despite the indicated limitations, this innovative systematic review offers an overview of saliva and its potential role as a source of biological biomarkers that may be useful for diagnosing or monitoring patients infected with SARS-CoV-2, especially considering the many advantages offered by this fluid. Considering the worldwide disorder caused by this virus and the need to detect infected patients, especially asymptomatic patients who could be unknowingly spreading the virus, the alternative of salivary collection, which could be carried out by the patient, reducing the risk of contagion in health care, proved to be sufficiently specific and sensitive (28) to be used in the general population. In addition, techniques such as immunoassays applied to saliva would allow for greater epidemiological control by being able to detect various sources of infection at the same time. Although the evaluated studies offer promising results, additional evidence is needed to confirm the efficacy of saliva as a sample fluid for diagnosing COVID-19. It is important to note that not only saliva could be useful as a complementary method in the diagnosis of COVID-19. SARS-COV-2 infection can cause oral manifestations (as oral vesiculobullous lesions) (35,36), highlighting the role of dental surgeons in the diagnostic process.
